# The Impact of Carbohydrate Quality on Dental Plaque pH: Does the Glycemic Index of Starchy Foods Matter for Dental Health?

**DOI:** 10.3390/nu13082711

**Published:** 2021-08-06

**Authors:** Fiona S. Atkinson, Jouhrah Hussain Khan, Jennie C. Brand-Miller, Joerg Eberhard

**Affiliations:** 1 Charles Perkins Centre, School of Life and Environmental Sciences, The University of Sydney, Sydney, NSW 2006, Australia; fiona.atkinson@sydney.edu.au (F.S.A.); jennie.brandmiller@sydney.edu.au (J.C.B.-M.); 2 Charles Perkins Centre, the School of Dentistry, The University of Sydney, Sydney, NSW 2006, Australia; jkha6470@uni.sydney.edu.au

**Keywords:** dental plaque pH, glycemic index, dental caries, carbohydrates

## Abstract

Sugary carbohydrate foods have long been associated with increased risk of dental caries formation, but the dental health impact of starchy carbohydrates, particularly those with a high glycemic index (GI), has not been well examined. Aim: To investigate the effect of different starchy foods varying in their GI, on acute changes in dental plaque pH. Methods: In a series of sub-studies in healthy adults, common starchy carbohydrate foods, including white bread, instant mashed potatoes, canned chickpeas, pasta, breakfast cereals, white rice, and an oral glucose solution were consumed in fixed 25 g available carbohydrate portions. The change in dental plaque pH was assessed postprandially over 1 h and capillary plasma glucose was measured at regular intervals over 2 h. Results: Higher GI starchy foods produced greater acute plaque pH decreases and larger overall postprandial glucose responses compared to lower GI starchy foods (white bread compared with canned chickpeas: −1.5 vs. −0.7 pH units, *p* = 0.001, and 99 ± 8 mmol/L min vs. 47 ± 7 mmol/L min, *p* = 0.026). Controlling for other food factors (food form and nutritional composition), lower GI versions of matched food pairs produced smaller plaque pH excursions compared to higher GI versions of the same food. Using linear regression analysis, the GI value of starchy carbohydrate foods explained 60% of the variation in maximum plaque pH nadir and 64% of the variation in overall acute dental plaque pH excursion (*p* < 0.01). Conclusion: The findings imply that starchy foods, in particular those with a higher GI, may play a role in increasing the risk of dental caries.

## 1. Introduction

Dental caries, widely known as tooth decay, is the most common non-communicable disease worldwide [1,2]. Dental caries results from carbohydrate fermentation by acid-producing bacteria within the dental biofilm. Bacteria present in the plaque ferment dietary carbohydrates, particularly sucrose, into acids that then cause a decrease in plaque pH adjacent to the tooth surface leading to demineralization of the tooth hard substances, e.g., enamel, dentine and cementum [3]. A decline in pH level below 5 in the tooth biofilm results in enamel demineralization [4], but dental caries etiology and onset is a complex process and simply consuming carbohydrates does not necessarily result in the development of caries. The formation and progression of dental caries is influenced by the presence of certain oral microorganisms, consumption of free sugars, development of enamel and cementum defects, saliva buffering capacity, socio-economic conditions, and oral hygiene practices [4,5]. Consumption frequency, texture, and duration of exposure to different carbohydrates are important factors that also influence dental caries formation. In general, foods that cause a larger decrease in plaque pH have a greater cariogenic potential [6,7,8] and therefore measuring plaque pH is considered a valid method to assess the capacity of different foods and beverages to increase the risk of dental caries.

The detrimental impact of sugary foods, such as carbonated beverages, fruit juices and sweet or sticky snack foods, on dental health has been widely studied. Typically, these products produce a large drop in dental plaque pH [7,8,9,10,11,12,13,14,15]. However, some beverages such as malted milk-based drinks produced an acute increase in plaque pH [10,12] and may help to prevent caries formation. Studies examining the potential impact of starchy carbohydrates on dental caries risk are more limited. In previous studies, different breads were found to produce variable decreases in plaque pH [16,17,18] and it has been hypothesized that they may contribute to higher dental caries risk. However, methodological variations amongst studies, in particular the amount of carbohydrate tested and the assessment method for plaque pH drop, restricts comparison and interpretation of results between studies.

Starch digestion commences in the oral cavity during mastication and mixing with α-amylase in saliva. Studies of tooth demineralization indicate that cooked wheat starch can be rapidly broken down to maltose by α-amylase in saliva [19]. Similarly, up to 50% of the starch in bread is reportedly hydrolyzed within 30 s of chewing and mixing with saliva in the mouth [20]. Traditionally, carbohydrates were classified based on their physical and chemical structure into ‘simple’ sugars or ‘complex’ carbohydrates [21]. However, this classification does not accurately reflect the speed of digestion and absorption of different types of carbohydrate in the oral cavity or digestive system. A systematic review of 28 studies concluded that only rapidly digested starch significantly increases caries risk, but the data were graded low quality [22].

The notion that complex carbohydrates are always slowly digested and absorbed is a common misconception. Ranking carbohydrate foods based on their physiological response in the body, known as the glycemic index, was introduced in the literature 40 years ago [23]. This is a measure of carbohydrate quality that ranks the blood-glucose raising potential of different foods, gram-for-gram of carbohydrate. Some foods may be more slowly digested and absorbed producing a lower GI, either because they contain sugars that are inherently less glycemic or the starch present is more resistant to enzyme digestion [24].

Limited studies have explored the influence of starchy carbohydrates on dental plaque pH. To our knowledge, no published studies have simultaneously investigated the impact of starch digestibility and carbohydrate quality, by investigating foods varying in their GI, on the impact of dental plaque pH. Therefore, the aim of our study was to examine the effect of different starchy carbohydrate foods, varying in their GI, on acute changes in dental plaque pH. In order to address some of the methodological variations in published studies, we also investigated the influence of carbohydrate dose and conducted studies where both the carbohydrate dose and the food texture were controlled to explore the impact of GI on dental plaque pH. We hypothesized that foods with a higher GI, which are more rapidly digested, would produce a larger decrease in dental plaque pH than lower-GI foods.

## 2. Materials and Methods

The study protocol was approved by the Human Research Ethics Committee of the University of Sydney (Protocol: 2017/801). All participants gave written, informed consent before participating in the study. The overall study design adhered to the standardized GI testing methodology [25] with the addition of the collection of dental plaque samples to allow for the simultaneous assessment of acute changes in dental plaque pH and postprandial glucose responses. The investigation was divided into three sub-studies to answer 3 specific research questions related to carbohydrate consumption and the potential impact on dental plaque pH. In the first study, the impact of carbohydrate dose on acute changes in dental plaque pH was investigated. In the second, carbohydrate amount was controlled but a range of different starchy foods was tested. In the third study, the food form, texture and carbohydrate amount were controlled by comparing results within pairs of the same food type differing in GI. The study was registered with the Australian New Zealand Clinical Trials Registry (ANZCTR: 12621000570886).

### 2.1. Participants and Study Design

Healthy adults aged 18–65 years, non-smoking, without any underlying health conditions or dental disease were recruited from the staff and student population of the University of Sydney. Individuals with food allergies, impaired glucose tolerance, those regularly taking prescription medications other than standard oral contraceptives, or individuals with orthodontic appliances were excluded. Throughout the study, participants maintained their usual diet and exercise habits and were instructed to avoid consuming legumes and alcohol for 24 h before each test session. They were required to consume a high-carbohydrate, low-fat evening meal and fast for 10–12 h overnight before each test session. In addition, they were asked to refrain from brushing their teeth and performing any oral hygiene the night before a test and on the morning of a test session.

### 2.2. Test Foods

In Study 1, a glucose solution containing either 25 or 50 g of available carbohydrate dissolved in 250 mL water (Table 1) was studied to investigate the impact of different carbohydrate amounts on acute changes in dental plaque pH. Each glucose solution was tested in triplicate in random order in each participant. Participants completed 6 test sessions, with consecutive sessions separated by at least 1 day.

In Study 2, four different starchy carbohydrate foods (canned chickpeas, boiled penne pasta, white bread, and instant mashed potatoes prepared with water) representing a wide range of basic carbohydrate foods with known GI values were compared with a reference glucose solution (Table 1). All four test foods and the reference solution were consumed in a portion containing 25 g available carbohydrate. The reference glucose solution was tested twice by each participant at the start and end of the study. Each of the four foods were consumed on one occasion in random order, with consecutive sessions separated by at least 1 day.

In Study 3, three different types of food-pairs were tested: two white rice, two white breads and two breakfast cereals, along with a reference glucose solution (Table 1). Within each pair, one food had a lower GI than the other, but were similar in texture, processing/cooking method, and food form. All test portions contained 25 g available carbohydrate, with a similar ratio of starch and sugar within each food pair. A qualified dentist collected and measured the plaque samples (J.K.) and was blinded to the differences in GI and nutritional information. All test foods in Study 2 and 3 were freshly prepared according to manufacturers’ instructions and without additional ingredients.

### 2.3. Dental Sampling and pH Measurement

Upon arrival, a dental plaque sample was collected in the fasted state using a sterile plastic dental probe (SI551ST Periodontal 3-Piece Examination Kit, MDDI, Kunda Park, Australia) with a metal tip designed to harvest supragingival plaque from the mesial, distal, buccal, and palatal/lingual tooth surfaces. A sufficient amount of plaque (~1 mm from the tip of the probe) was sampled at each time point. The aliquot collected was then suspended in 25 µL of distilled water in a 2.5 mL tube and vortexed for 20 s. The pH was assessed immediately using a calibrated pH electrode (SevenCompact^TM^ pH/Ion S220, Greifensee, Switzerland). Before each measurement, the electrode was rinsed in distilled water, dried, and calibrated to pH 7.0. For Study 1, additional dental plaque samples were collected 2, 12 and 32 min after complete consumption of the test beverage. A different quadrant of the oral activity was randomly selected for each plaque sampling. For Studies 2 and 3, dental plaque samples were collected at 12, 22 and 62 min after completion of eating.

In Studies 2 and 3, a fasting saliva sample (>1 mL) was collected using a Salivette tube (Sarstedt AG & Co, Nümbrecht, Germany) for the assessment of saliva buffering capacity using Ericsson’s method [26]. Stimulated saliva was collected for 5 min, after which the collection tube was centrifuged at 2000× *g* for 2 min at 20 °C. Saliva was then pipetted into an uncoated tube and stored at −30 °C. To measure buffering capacity, 1 mL of thawed saliva was mixed with 3 mL HCl (0.5%) and one drop of 97% 2-Octanol (Sigma-Aldrich, Bayswater, Australia) to prevent foaming. The solution was then mixed for 20 min in a suspension mixer (Selby, Melbourne, Australia) after which pH was measured using a calibrated electrode (SevenCompact^TM^ pH/Ion S220, Greifensee, Switzerland).

### 2.4. Blood Sampling and Plasma Glucose Measurement

For Studies 2 and 3, capillary finger-prick blood samples (>0.5 mL) were collected from a warmed hand twice in the fasted state (−5 and 0 min) and then at regular intervals during the postprandial period for the following 2 h (15, 30, 45, 60, 90 and 120 min after eating had commenced). Each sample was centrifuged at 10,000× *g* for 45 s immediately after collection and the plasma layer stored at −30 °C for subsequent analysis. Plasma glucose concentration was measured in duplicate using a glucose hexokinase assay (Beckman Coulter Inc., Carlsbad, CA, USA) on an automatic centrifugal spectrophotometric clinical chemistry analyzer (Beckman Coulter AU480^®^, Beckman Instruments Inc., Carlsbad, CA, USA).

### 2.5. Data Analysis

The incremental area above the plaque pH response curve (iAAC) or incremental area below the plasma glucose response curve (iAUC) was calculated using the trapezoidal rule, ignoring any area above the baseline line (plaque pH) or below baseline (plasma glucose). GI values for the test products were determined for each participant by expressing their glucose iAUC response for the test food relative to their glucose iAUC response to the reference food [25]. Standard parametric statistical tests (Analysis of Variance) with Bonferroni adjustment were performed using IBM^®^ SPSS^®^ Statistics software (version 24) to determine significant differences in pH (iAAC) and glycemia (iAUC). ANOVA was used to assess the effect of different foods at the maximum decrease in plaque pH (12 min in Study 1 and 22 min in Studies 2 and 3). Pearson correlation coefficients were calculated to test the association between dental plaque pH parameters and GI. *p* < 0.05 was considered statistically significant. Data are shown as mean ± standard error of the mean unless otherwise stated.

## 3. Results

### 3.1. Study 1: Impact of Carbohydrate Dose on Acute Changes in Dental Plaque pH

**Figure 1:**
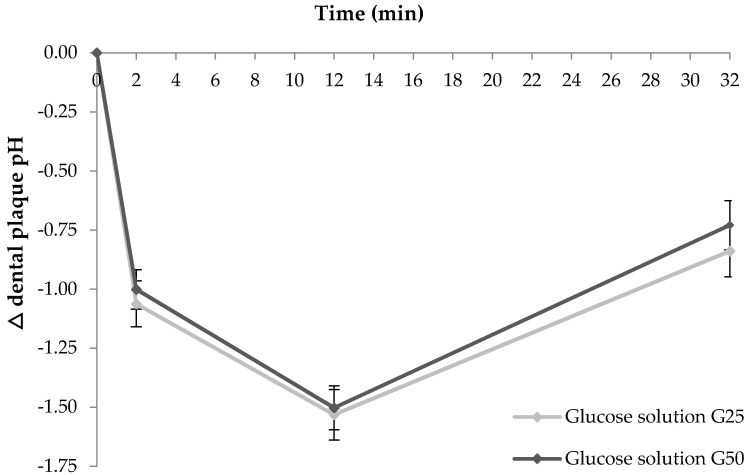
Change in postprandial dental plaque pH in response to two different doses of carbohydrate in 8 healthy adults. Data are mean ± standard error mean (SEM), *n* = 24 for the three repeated tests in each of the 8 participants for the glucose solution containing either 25 g available carbohydrate (shown in pale grey) or 50 g available carbohydrate (shown in dark grey).

Eight healthy individuals (one male and seven females, mean age 32.9 ± 11.0 y) participated. The two glucose solutions differing in their carbohydrate dose produced a similar overall acute postprandial change in dental plaque pH (Figure 1). Both doses (25 g and 50 g available carbohydrate) produced similar maximum decreases in dental plaque pH at 12 min (−1.53 and −1.50 pH units, respectively, *p* = 0.83). No significant difference between the 25 g and 50 g available carbohydrate glucose solutions was observed (iAAC: 35 ± 3 vs. 38 ± 3 pH units/min, respectively, *p* = 0.56).

### 3.2. Study 2: Impact of Carbohydrate Quality (GI) on Acute Changes in Dental Plaque pH

**Figure 2:**
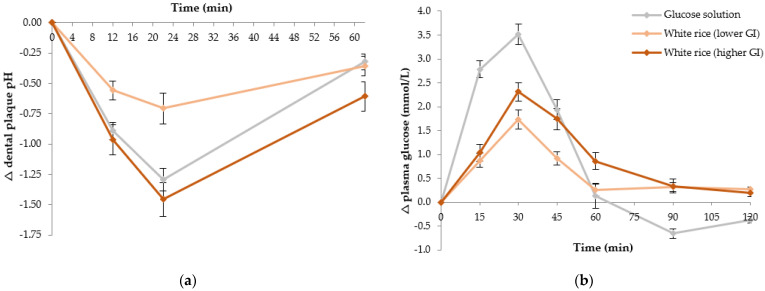
Impact of 25 g available carbohydrate portions of different carbohydrate foods, varying in GI, on dental plaque pH in 12 healthy participants (**a**) Change in postprandial dental plaque pH over 62 min; (**b**) Change in plasma glucose concentration over 120 min. Data are mean ± standard error mean (SEM), *n* = 24 for the two repeated glucose solution tests (shown in grey), and *n* = 12 for each of the four test foods: canned chickpeas (shown in green), penne pasta (shown in purple), white bread (shown in red) and instant mashed potatoes (shown in yellow).

Fixed 25 g available carbohydrate portions were used based on the findings in Study 1. Twelve healthy individuals (four males and eight females, mean age 24.9 ± 5.9 y, mean BMI 23.1 ± 2.9 kg/m^2^) participated. Figure 2 shows the overall acute response changes in dental plaque pH and postprandial plasma glucose produced by the four starchy test foods and the reference food. The maximum decrease in plaque pH level occurred at 22 min (Figure 2a), with the white bread producing the greatest decrease of −1.5 units compared to baseline. The canned chickpeas produced the smallest decrease (nadir) at 22 min of −0.7 pH units, which was significantly smaller than the glucose solution (*p* = 0.029) and the white bread (*p* = 0.001). Similarly, the canned chickpeas produced a significantly lower iAAC response compared to the white bread (30 ± 4 pH units/min vs. 63 ± 6 pH units/min, respectively, *p* = 0.004).

The glucose solution (the reference food) produced a larger increase in postprandial glycemia from 0 to 30 min compared to the chickpeas (3.62 mmol/L vs. 0.96 mmol/L, *p* < 0.001), penne pasta (3.62 mmol/L vs. 1.37 mmol/L, *p* < 0.001) and white bread (3.62 mmol/L vs. 2.19 mmol/L, *p* < 0.001) (Figure 2b). No significant difference in absolute change in plasma glucose from 0 to 30 min was observed between the glucose solution and the instant mashed potatoes. The canned chickpeas, pasta and white bread all produced a significantly smaller absolute change in plasma glucose from 0 to 30 min compared to the instant mashed potatoes (*p* < 0.001). The absolute change from 0 to 30 min for chickpeas was also found to be significantly lower than that produced by the white bread (*p* = 0.001).

The overall plasma glucose response (iAUC) produced by the chickpeas (47 ± 7 mmol/L min) was significantly lower than the overall postprandial responses produced by the glucose solution (135 ± 11 mmol/L min) and instant mashed potatoes (127 ± 13 mmol/L min) (both *p* < 0.001), and the white bread (99 ± 8 mmol/L min) (*p* = 0.026) (Figure 2b). Similarly, the GI value for chickpeas (35 ± 4) was significantly smaller than the GI values observed for the white bread (77 ± 5), instant mashed potatoes (96 ± 5) and glucose solution (assigned GI = 100 by definition) (all *p* < 0.001). The overall plasma glucose response (iAUC) produced by the pasta was lower than the glucose solution (61 ± 7 mmol/L min vs. 135 ±11 mmol/L min, *p* < 0.001) and the instant mashed potatoes (61 ± 7 mmol/L min vs. 127 ± 13 mmol/L min, *p* = 0.002) (Figure 2b). Similarly, the GI value for pasta (47 ± 4) was significantly smaller than the average GI values produced by the white bread (77 ± 5), mashed potatoes (96 ± 5) and the glucose solution (all *p* < 0.001). The GI value for the white bread (77 ± 5) was also lower than that of the glucose solution (*p* < 0.001) and the mashed potato (96 ± 5) (*p* = 0.006).

### 3.3. Study 3: Impact of GI in Paired Foods on Acute Changes in Dental Plaque pH

**Figure 3:**
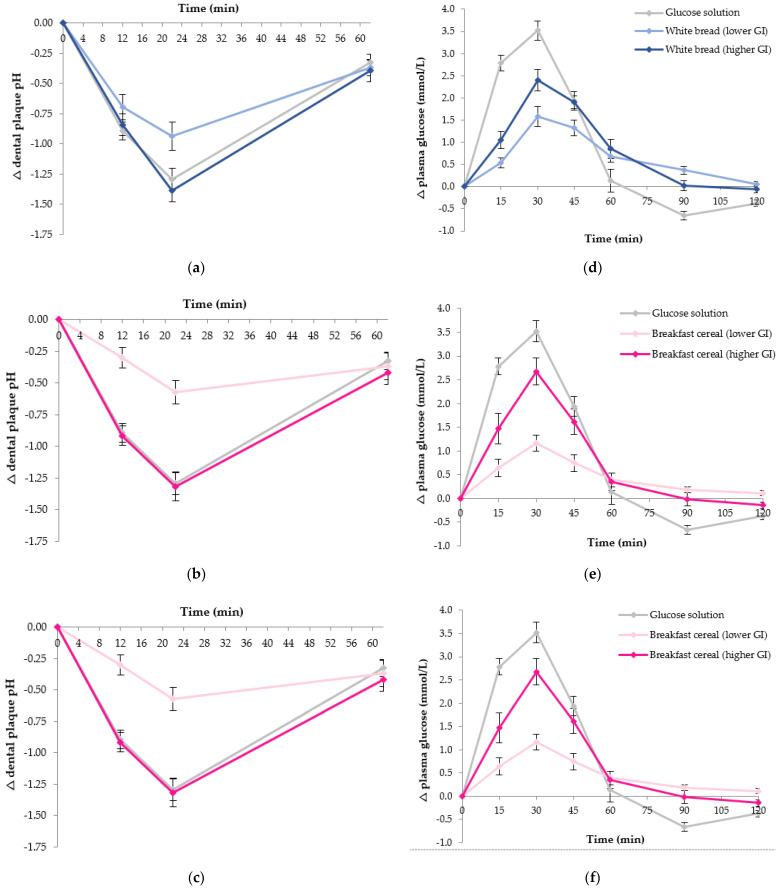
Impact of 25 g available carbohydrate portions of different carbohydrate food pairs, varying in GI, on dental plaque pH in 12 healthy participants (**a**) Change in postprandial dental plaque pH over 62 min following consumption of a lower GI rice (shown in pale orange) and a higher GI rice (shown in dark orange); (**b**) Change in postprandial dental plaque pH over 62 min following consumption of a lower GI white bread (shown in pale blue) and a higher GI white bread (shown in dark blue); (**c**) Change in postprandial dental plaque pH over 62 min following consumption of a lower GI breakfast cereal (shown in pale pink) and a higher GI breakfast cereal (shown in dark pink); (**d**) Change in plasma glucose concentration over 120 min following consumption of two types of rice varying in GI; (**e**) Change in plasma glucose concentration over 120 min following consumption of two breads varying in GI; (**f**) Change in plasma glucose concentration over 120 min following consumption of two breakfast cereals varying in GI. Data are mean ± standard error mean (SEM), *n* = 24 for the two repeated glucose solution tests (shown in pale grey), and *n* = 12 for each test food.

Twelve healthy individuals (four males and eight females, mean age 25.8 ± 3.3 y, mean BMI 23.3 ± 2.9 kg/m^2^) participated. Figure 3 shows the overall acute postprandial response changes in dental plaque pH and plasma glucose produced by the three pairs of carbohydrate foods: white rice, white bread and breakfast cereals. The reference food (glucose solution) is shown on each figure panel in grey for comparison.

The lower GI white rice produced a smaller plaque pH fall at 22 min compared to the higher GI rice (*p* < 0.001) and reference glucose solution (*p* = 0.003) (Figure 3a). The integrated change in plaque pH was also smaller (31.0 pH units/min vs. 59.2 pH units/min, *p* = 0.004). Notably, there was no significant difference in maximum nadir or overall plaque pH change between the higher GI rice and the reference glucose solution. Numerically, the lower GI white bread showed a smaller pH drop (nadir) at 22 min and a smaller overall pH response over 62 min compared to the higher GI bread and the reference food, although these differences did not reach statistical significance (Figure 3b). The lower GI breakfast cereal produced a smaller plaque pH nadir at 22 min relative to both the higher GI breakfast cereal (*p* = 0.001) and the reference glucose solution (*p* < 0.001) (Figure 3c). A smaller overall change in plaque pH was observed for the lower GI breakfast cereal compared to the higher GI variant (25.0 pH units/min vs. 51.4 pH units/min, *p* = 0.010), and compared to the glucose solution (25.0 pH units/min vs. 48.7 pH units/min, *p* = 0.006).

The lower GI rice produced a smaller change in peak in plasma glucose at 30 min compared to the reference glucose solution (*p* < 0.001) and a significantly lower GI compared to both the higher GI rice (55 ± 4 vs. 84 ± 5, *p* < 0.001) and the glucose solution (*p* < 0.001) (Figure 3d). The lower GI white bread showed a significantly smaller change in peak plasma glucose between 0–30 min compared to the reference food (*p* < 0.001) and a smaller GI compared to the higher GI white bread (60 ± 4 vs. 82 ± 5, *p* = 0.003) and the reference food (*p* < 0.001) (Figure 3e). The lower GI breakfast cereal showed a smaller change in peak in plasma glucose at 30 min and lower overall plasma glucose iAUC response over 120 min compared to both the higher GI cereal (*p* = 0.001 and *p* = 0.025, respectively) and the reference glucose solution (both *p* < 0.001). A significantly lower GI was observed for the lower GI breakfast cereal compared to both the higher GI breakfast cereal (43 ± 4 vs. 77 ± 5, *p* < 0.001) and the glucose solution (*p* < 0.001) (Figure 3f).

The average saliva buffering capacity for the participants in Studies 2 and 3 was 3.27 ± 0.57 (SD), with all subjects producing pH values the very low to normal classification.

### 3.4. Association between GI and Change in Dental Plaque pH

**Figure 4:**
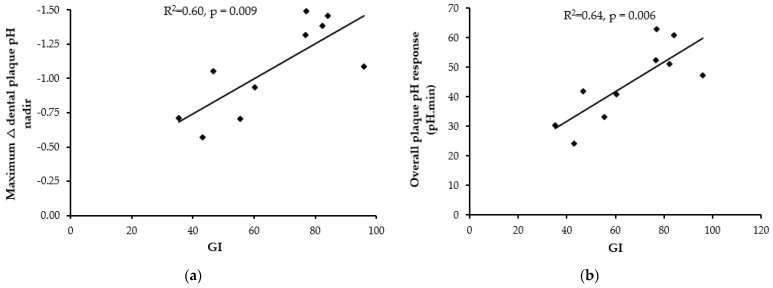
Linear correlation between GI of starchy foods tested in sub-study 2 (*n* = 4 foods) and sub-study 3 (*n* = 6 foods) and maximum nadir decrease in dental plaque pH at 22 min (**a**) or overall plaque pH response over 62 min (**b**). Each data point represents the average response in 12 healthy participants.

The average GI values produced by the four starchy foods tested in Study 2 and the six starchy foods tested in Study 3 were strongly correlated with the maximum change in dental plaque pH (Figure 4a) and the overall dental plaque pH change over 62 min (iAAC response) (Figure 4b). Similarly, there was a strong correlation between the GI values and the change in plaque pH measured as either the maximum decrease at 22 min (R^2^ = 0.60, *p* = 0.009) or the overall change in dental plaque pH over 62 min (R^2^ = 0.64, *p* = 0.006).

## 4. Discussion

In the present studies, we demonstrate that the GI of starchy carbohydrate foods is a good predictor of the acute changes in dental plaque pH after consumption, explaining more than 60% of the variation in pH. Controlling for food type, we also showed that breads, breakfast cereals and rice with a higher GI value produce a larger decrease in dental plaque pH than their counterparts with a lower GI. Notably, higher GI breads, rice, breakfast cereals, and potatoes elicited comparable reductions in plaque pH to a 10% glucose solution. These findings should not be surprising because the same mechanisms that make starch easily digestible by humans also make them rapidly digestible by microorganisms in dental plaque. Our findings also imply that both ‘simple’ and ‘complex’ carbohydrate types possess similar abilities to increase the risk of dental caries. To our knowledge, this is the first systematic comparison of high and low GI starchy foods on changes in plaque pH.

In Study 1, we examined the impact of carbohydrate dose on acute changes in dental plaque pH. The two different carbohydrate doses, 25 g or 50 g of glucose sugar (10% and 20% solutions) produced similar maximum decreases in plaque pH and consistent overall plaque pH excursions over 62 min. Published data have not typically controlled the carbohydrate test portion either within or between studies, with test portions varying from as low as 2 g up to 25 g of carbohydrate, limiting assessment of the cariogenic potential of different carbohydrate foods between studies [7,10,14,16,17,18]. Furthermore, carbohydrate test portions used in previous studies may not represent realistic amounts of foods typically consumed, for example 10 mL yoghurt [27], 10 g breakfast cereal [13], or 15 mL soft drink [7], potentially restricting the real-world translation and applicability of results. Typically, a meal contains 50 to 100 g of carbohydrate, with different foods contributing varying proportions. We observed that a 25 g available carbohydrate portion was sufficiently large to significantly impact dental plaque pH and may represent both a realistic approximation for a snack or meal and a carbohydrate dose likely to produce a maximal change in plaque pH.

In Study 2, the different starch-containing carbohydrate foods were chosen to represent common carbohydrate staples that vary in their GI, texture, and nutritional composition. As hypothesized, foods that produced lower postprandial glycemic responses also elicited smaller dental plaque pH excursions. The smaller plaque pH nadir may reflect the slower rate of digestion of carbohydrates observed with lower GI foods [23,28,29], resulting in less acid production after fermentation by oral bacteria. The intact nature of the whole canned chickpeas [30] and the protein-starch matrix found in pasta [31] are both factors known to create a physical barrier delaying enzymatic digestion of starch. Secondly, food form may also play a role with the dry or crumbly texture of the canned chickpeas and pasta less likely to stick to tooth surfaces for bacterial fermentation compared to the white bread or instant mashed potatoes. Texture has been shown to significantly affect plaque pH changes and dental caries formation with thick or sticky foods having greater potential to adhere to tooth surfaces for an extended period [27]. Thirdly, the level of food processing may partly explain the results [32]. More highly gelatinised foods, such as instant mashed potatoes, have major alterations in the structural and physical properties of the starch making them more susceptible to degradation, compared to less processed foods, such as chickpeas. It has been previously demonstrated that more highly processed versions of grain foods had higher GI values, which correlated to the degree of starch gelatinization [33].

Although Study 2 showed that different starchy foods produced differences in acute changes in dental plaque pH, it was not possible to isolate whether GI was a specific influencing factor as opposed to variations in physical form and texture, or differences in macronutrient composition. Therefore, three food pairs were examined in Study 3. The nutritional composition, texture, processing method, and portion size within each food pair were designed to be as closely matched as possible. The lower GI version within each pair of rice, white bread or breakfast cereal produced a smaller nadir in plaque pH and a lower overall pH excursion compared to the higher GI version of the same food type.

One of the potential mechanisms underlying this result is that higher GI foods, which a more rapidly digested, may produce greater amounts of preferred substrate for oral bacteria resulting in greater acid production. Intraoral pH is significantly affected by an acidic environment which is influenced not only by intrinsic factors but also by extrinsic factors, such as the type of food consumed [34]. This result provides evidence that higher GI foods, which a more rapidly digested and produce a larger rise in glucose response, have a greater impact on dental plaque pH and potentially lead to an increased risk of dental caries formation in the longer term.

By design, we included starchy foods with a range of GI values and simultaneously assessed postprandial glucose responses and changes in dental plaque pH. A significant proportion of the variation in either the maximum plaque pH nadir or the overall acute pH excursion was explained by the GI of the foods (60% and 64%, respectively). To our knowledge our study findings are novel, showing a significant association between the GI value of carbohydrates and dental plaque pH. There is some support for our finding in the literature. By assigning published GI values [35] to foods tested in other studies measuring dental plaque pH changes, we can see a similar anecdotal trend with lower GI foods showing smaller pH excursions, for example with whole fruits [36] or sports beverages [37].

The strengths and limitations of our studies should be noted. We reduced variation between and within individuals by testing multiple groups of participants, and used a reference food which was tested twice by each participant. We assessed glycemic responses and pH changes in plaque simultaneously and confirmed the known GI values of the food tested. The sampling/scraping technique we used for plaque pH measurement has shown the smallest plaque pH drop after food ingestion, compared to the micro-touch or telemetric methods [17]. A limitation is that we did not record usual dietary intake or the Decayed, Missing and Filled Teeth (DMFT) index for the study participants. The changes observed in the present study may therefore represent a conservative estimation of the true magnitude of the acute plaque pH changes in response to starchy foods varying in GI. Buffering capacity of saliva, which assesses the ability of saliva to counteract acidic changes in the mouth, is known to affect plaque pH and, in turn, caries formation [38,39]. In our studies, 94% of participants had either very low or low buffering capacity in their fasting saliva sample. The finding of low buffering capacity amongst our young adult participants may indicate measurement error or a bias in sampling as epidemiological studies have shown that about 33% of adults <35 years have a low buffering capacity of saliva [40]. Due to the similar intra-individual buffering capacity and the collection of only one fasted sample, we did not include this factor in our statistical analysis. In future studies, the collection of multiple saliva samples would be beneficial to thoroughly investigate the influence of buffering capacity on acute dental plaque pH in response to foods.

## 5. Conclusions

Our findings imply that the GI of foods is not just relevant to the risk of chronic diseases such as type 2 diabetes and cardiovascular disease, but also to dental caries. Starchy foods are often ignored when dentists counsel patients about oral care and dental caries etiology. Oral health education on dietary intake has traditionally focused on reducing sugary foods, in particular those containing sucrose, to prevent tooth decay. The results of our studies show that starchy foods, particularly those that are more rapidly digested in the oral cavity, have a similar or greater potential than sugary foods to drop plaque pH, and may therefore pose an increased risk of dental caries. Individuals with high rates of dental caries or poor oral health may benefit from consuming diets containing carbohydrates that are slowly digested and absorbed, such as low-GI foods, to help alleviate symptoms or reduce the future incidence of dental caries.

## Figures and Tables

**Table 1:** Nutritional information for the foods tested in each sub-study.

Study	Food	Weight (g)	Energy (kJ)	Protein (g)	Fat (g)	Available Carbohydrate (g)	Starch (g)	Sugar (g)	Fiber (g)
1	Glucose solution G25	25.7	436	0	0	25	0	25	0
	Glucose solution G50	51.4	873	0	0	50	0	50	0
2	Canned chickpeas	131.5	751	9.4	2.7	25	24.4	0.6	7.8
	Penne pasta	34.7 ^1^	531	4.1	0.6	25	24.2	0.8	1.0
	White bread	58.5	585	4.3	1.8	25	23.0	2.0	4.1
	Instant mashed potatoes	37.1 ^1^	602	3.3	2.7	25	23.3	1.7	-
3 ^2^	White rice (lower GI)	69.5	553	2.6	1.8	25	24.7	0.3	1.7
	White rice (higher GI)	73.5	574	3.2	2.2	25	24.7	0.3	0.9
	White bread (lower GI)	60.5	604	4.9	1.3	25	23.4	1.6	5.6
	White bread (higher GI)	61.8	599	4.6	1.5	25	23.2	1.8	5.1
	Breakfast cereal (lower GI)	38.2	581	3.7	1.0	25	20.2	4.8	7.0
	Breakfast cereal (higher GI)	32.8	528	2.2	1.2	25	20.3	4.7	2.3

^1^ Dry weight of food. ^2^ During Study 3 conduction, foods were simply labelled as Rice A, Rice B, Bread A, Bread B, Breakfast cereal A and Breakfast cereal B. The designation of A vs. B was different within each food pair and did not correspond to low and high GI.

## Data Availability

The data presented in this study are available on request from the corresponding author. The data are not publicly available due to privacy restrictions.
